# Intracranial Inflammatory Myofibroblastic Tumor: A Review of 49 cases

**DOI:** 10.4322/acr.2021.254

**Published:** 2021-03-12

**Authors:** Deepika Phogat, S.G.S Datta, Mukul Bajpai, Swayam Tara, Sunil Kumar Ganti

**Affiliations:** 1 151 Base Hospital, Department of Pathology, Guwahati, Assam, India; 2 151 Base Hospital, Department of Neurosurgery, Guwahati, Assam, India; 3 151 Base Hospital, Department of Anesthesia, Guwahati, Assam, India

**Keywords:** Granuloma, Plasma Cell, Meningioma, Seizures, Magnetic ResonanceImaging, Meningeal Neoplasms

## Abstract

Inflammatory Myofibroblastic Tumor (IMT) is a rare pathologic entity that was first described in 1973. This lesion is most commonly found in the lungs, but other organs’ involvement has also been reported. Intracranial location of Inflammatory Myofibroblastic Tumor is rare, and the first case was reported in 1980. An intriguing fact about the intracranial IMT is its resemblance with meningioma on clinical presentation and neuroimaging. We came across a case of intracranial Inflammatory Myofibroblastic Tumor (IIMT) in a 27-year-old male who presented with recurrent episodes of seizures and was diagnosed as meningioma on neuroimaging. The lesion did not subside with medical management and kept on progressing in size. The patient had to undergo surgery, and diagnosis of Inflammatory Myofibroblastic Tumor was ascertained on histopathology. This ‘surprise’ diagnosis prompted us to review the literature on all cases of IIMTs reported to date to better understand the entity and its implications. In this review article, we present our observations regarding various studied parameters, including patient profile, clinical presentation, site of involvement, focality of the lesion, special associations, and lines of management of the 49 published cases of IIMTs.

## INTRODUCTION

Inflammatory Myofibroblastic Tumor (IMT) is a rare enigmatic pathologic entity with poorly understood pathogenic mechanisms and progression. It is characterized by benign proliferation of inflammatory cells and is known by multiple synonyms such as (i) plasma cell granuloma, (ii) inflammatory pseudotumor, and (iii) cellular inflammatory pseudotumor. This entity was first described by Bahadori and Liebow in 1973,^1^ and has been commonly reported in the lungs and the upper respiratory tract.

The intracranial location of this lesion is rare and was first reported by S G West et al.,^2^ in 1980.

Though in most of the cases, the lesions are unifocal, multifocal involvement of intracranial and extracranial sites has also been reported in some studies.^3^^,^^4^

The common presenting complaints of the IIMT are headache, seizures, ataxia, and visual disturbances. Cranial magnetic resonance imaging (MRI) shows enhancing lesions commonly associated with dural attachment, which closely mimics neuroimaging findings of a meningioma. Histologically this lesion is characterized by proliferation of polyclonal plasma cells ascertained with kappa and lambda light chain, on immunohistochemistry.

Some studies have shown associations of IIMTs with polyclonal gammopathy, and one study has also reported an association with Epstein Barr Virus infection.^5^^,^^6^

We came across a case of IIMT is a 27-year-old previously healthy male who presented with episodes of generalized tonic-clonic seizures.

This case prompted us to research the literature on IIMTs to evaluate various parameters as patient profile, clinical presentation, site of involvement, focality of the lesion, special associations, and lines of management of IIMTs.

## CASE REPORT

A 27-year-old previously healthy male presented with episodes of generalized tonic-clonic seizures. There was no history of headache, visual disturbances, ataxia, or paresis. The results of routine laboratory investigations, including complete blood count, blood biochemistry, urinalysis, and electrocardiogram were within normal limits. Chest X-ray and abdominal ultrasound were normal. The cranial MRI revealed an intensely enhancing extra-axial dural-based lesion measuring 4.6 x 2.8 x1.1 cm in the left frontoparietal area with mild associated perilesional edema and no significant mass effect, suggestive of meningioma (Figures 11B).

**Figure 1:**
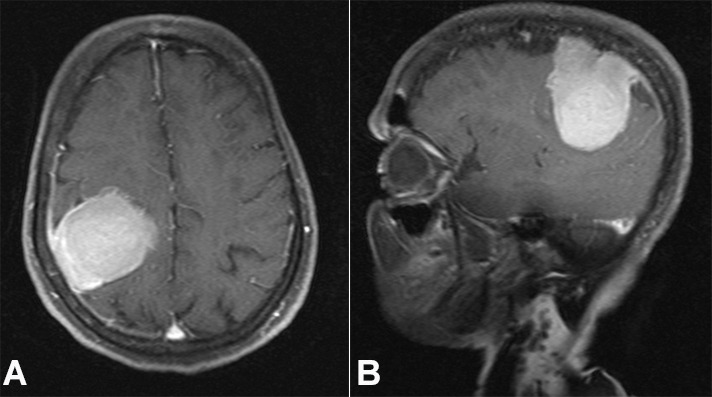
Brain MRI T1 weighted images post-contrast, **A –** axial plane, and **B** – sagittal plane, showing fat-saturated and avidly enhancing oval-shaped extra-axial lesion along the left frontoparietal convexity measuring 52mm in the longest dimension. The lesion shows a dural tail characteristic of meningioma.

The patient was continued on anti-epileptic medications with regular follow-ups. Surgical management was delayed in view of the small size of the lesion. While on medical management, the patient kept suffering recurrent episodes of generalized tonic-clonic seizures.

Brain MRI was repeated after 5 months of the initial neuroimaging, which revealed increased dimensions of the lesion - 5.2 x 3.0 x 1.1 cm (Figures 11B).

**Figure 2:**
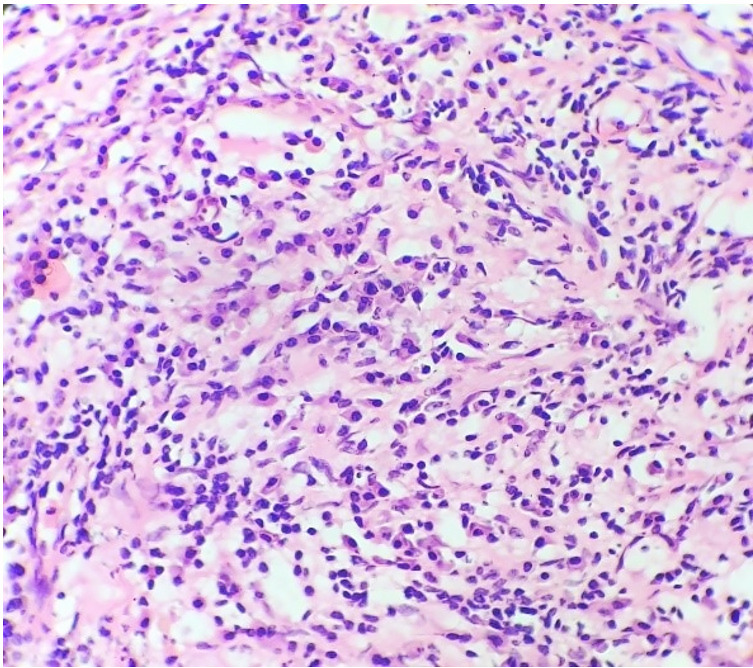
Histopathological examination with dense aggregates of inflammatory cells comprising predominantly of plasma cells (H&E, 40x).

The patient then underwent elective surgery, and the surgical specimen of the excised space-occupying lesion was analyzed. The external surface of the specimen was smooth and firm. The cut surface was homogenous and yellowish-white in appearance. No areas of hemorrhage or necrosis were identified on the gross examination. The sample was fixed in 10% formalin, embedded in paraffin, and stained with Hematoxylin &Eosin (H&E), Periodic Acid Schiff (PAS), Gram and Ziehl-Neelsen (ZN) Stains. Microscopic findings revealed a lesion composed of fibro-collagenous stroma with interspersed endothelium lined blood vessels. Dense aggregates of inflammatory cells comprising predominantly of plasma cells along with lymphocytes were noted in the perivascular areas within the stroma (Figure 2).

Interspersed histiocytes with round vesicular nuclei and foamy cytoplasm were also noted. Normal brain parenchyma was noted at the periphery.

No meningothelial cells or psammoma bodies were seen. No atypia, mitosis, or necrosis was noted. PAS and ZN stains were negative for microorganisms. Histomorphological features favored the diagnosis of Intracranial Inflammatory Myofibroblastic Tumor. Immunohistochemical tests were done to confirm the diagnosis and to rule out monoclonal plasma cell proliferation. Immunohistochemistry was performed on deparaffinized sections of each case with CD138 (Dako), Vimentin (Dako), SMA(Dako), S-100 (Dako), CD68(Dako), Anti Kappa (Biogenex), Anti Lambda polyclonal sera (Biogenex) using avidin biotin peroxidase complex. Plasma cells were positive for CD138 and showed equal presence of kappa and lambda positivity. No evidence of clonal plasma cells was noted. Vimentin was diffusely positive in the stroma, SMA was positive in blood vessels. Histiocytes were positive for S100 and CD68. The diagnosis was confirmed as Intracranial Inflammatory Myofibroblastic Tumor. Additional serological tests were done to rule out Epstein Barr Virus (EBV) as infective etiology. EBV IgM ELISA was <10U/ML (<20U/ML: negative). EBV IgM to Nuclear Antigen was 2.64u/ml (<8: negative). EBV IgG ELISA was 0.42(<1.00 Negative). This case prompted us to review the literature on cases of Intracranial Inflammatory myofibroblastic tumor.

In our pre-specified protocol, the keywords “intracranial inflammatory myofibroblastic tumor, intracranial plasma cell granuloma, inflammatory pseudotumor and cellular inflammatory pseudotumor” were used to gather the target papers for a systematic review of the literature. The databases Google Scholar, Research Gate, and Pub Med were used to search the articles published until October 2020. All types of articles, including case reports, case series, pictorial assays with titles that included the aforementioned keywords, were studied.

All articles were evaluated for variables, including the year of publication, the number of studies published, and the number of cases reported in these studies.

Other parameters evaluated were clinical profile including age and gender, the chief presenting complaint, site of the lesion, focality of the lesion, association of the lesion with the dura, special features or findings associated with the intracranial Inflammatory Myofibroblastic Tumor, and the management approach.

## RESULTS

After the review of literature, data on the published cases of intracranial inflammatory myofibroblastic tumors was compiled in chronological order (decade wise). (Table 1)

**Table 1:** Clinical characteristics of the IIMT retrieved from the literature

#	Ref	Gender/Age	Presentation	Site	DA	Associations	Treatment
1	^2^	M 17	Headache	L Posterior Fossa	+	PG	CSE
2	^7^	F 16	Headache	R Frontal lobe	+	-	PSE, steroids, RT
3	^8^	M 16	Headache	R Fronto-parietal lobe	+	-	CSE
4	^9^	F 36	Hemiparesis	Fourth ventricle	-	-	CSE
5	^10^	F 29	Seizure	R superior temporal gyrus	+	Necrosis	CSE
6	^5^	M 60	Seizure	R temporal lobe	+	PG	CSE
7	^11^	M 11	Headache	Frontal lobe	NA	-	CSE
8	^12^	M 40	Headache	R cavernous sinus	-	-	PSE, steroids
9		M 30	Blindness	L cavernous sinus	-	-	PSE, Steroids
10		F 11	Headache	Vermis cerebelli	-	-	CSE
11		M 56	Diabetes Insipidus, Headache	Pituitary stalk	-	-	CSE
12	^13^	F 60	Seizure	R middle cranial fossa	+	-	CSE
13	^6^	M 70	Headache, Seizure, Subcutaneous mass	R temporal lobe	+	EBV +, extracranial to intracranial spread	PSE, Steroids, RT
14	^14^	M 18	Epilepsy Bilateral ectopia lentis	L parietotemporal lobe	NA	Hypergammaglobulinemia, homocystinuria, thromboembolism	Diagnosed on autopsy
15	^15^	F 18	Headache	R temporal lobe	-	-	CSE
16	^16^	F 11	Seizure	R frontal lobe	+	-	CSE
17	^17^	F 34	L Ptosis, Transient oculomotor nerve palsy	Pituitary Stalk	+	-	CSE
18	^18^	F 35	Seizure	L parietal lobe	NA	-	CSE
19	^3^	M 34	Headache, Paraparesis	Cerebral, cerebellar, brain stem, intramedullary	NA	-	PSE, steroids, ATT
20	^19^	M 13	Seizure	R frontal lobe, lung	NA	Involvement of lung followed by brain 4 years later	CSE
21	^20^	F 44	Headache	R tentorium, Falx	+	-	PSE, steroids, RT
22	^21^	F 6	Headache, tinnitus	Cerebellopontine angle	NA	-	PSE, RT
23		M 41	Seizure	R occipital lobe	NA	-	Systemic chemotherapy
24		F 33	Hearing loss	Meninges	_+_	-	PSE, RT
25	^22^	M 13	Seizure	R frontal lobe	NA	-	CSE
26	^23^	M 70	Visual disturbance	Frontal lobe, third ventricle, Cranial base	NA	osteal erosion	Steroids, RT
27	^24^	F 22	Seizure	Temporo basal	+	-	PSE, Steroid
28	^22^	F 14	Headache, R acute otitis media	Cavernous sinus, R middle cranial fossa, Infratemporal fossa	NA	Raised IgM	Steroids, RT, 6MP, MTX
29	^25^	F 18	Seizure	L frontoparietal region	+	Extensive ossification	PSE
30	^26^	M 63	L hemiparesis	R frontal	NA	Multiloculated cystic morphology, + Colon cancer	PSE, RT
31	^27^	M 51	Headache	Para sagittal	+	Relapsing perichondritis	PSE, RT
32	^28^	F 58	Headache	L frontotemporal lobe	+	-	PSE, Steroid
33	^29^	F 44	Visual disturbance	R Tentorium	+	-	PSE, Steroids, RT
34	^30^	F 60	Blurred vision	Posterior fossa	+	Ig G4 -, ALK -	CSE, RT
35		F 52	R quadrantanopia	R ventricle	+	Ig G4 +, ALK -	CSE
36		M 45	hemiparesis	Frontal lobe	+	Ig G4 +, ALK -	CSE, Steroids
37		F 26	Headache	Fronto temporal	-	Ig G4 +, ALK -	CSE,Steroids
38	^31^	F 47	Seizures	L parietal lobe	-	-	CSE
39	^32^	M 59	Impaired vision	Intrasellar, Trans-sphenoidal	+	-	CSE, RT
40	^33^	M 47	Impaired vision, spastic quadriparesis	R cerebellopontine angle	+	-	CSE, Steroids, RT
41	^34^	F 47	1.Seizures	L parietal lobe	NA	-	CSE, steroids
42		M 56	L temporal headaches	L basal ganglia	NA	-	CSE, steroids
43	^35^	F 66	L sided hemiataxia	L cerebellar hemisphere	+	-	PSE, steroids, RT
							RIX
44	^36^	F 55	Headache, hearing loss	L temporal lobe	NA	4 Recurrences, correlation with ESR	PSEs, steroids, RT
45	^37^	M 52	Headache, blurred vision	Clivus	NA	-	CSE
46		F 55	Otalgia L ear with discharge	L nasopharynx and carotid space with osteolytic destruction	NA	IgG4 +	.RT
47	^4^	M 49	Gait ataxia	Multiple, Bifrontal, temporal	+	-	CSE
48	^38^	F 72	Headache	L cerebellar hemisphere	-	-	CSE
49	^39^	F 62	Seizures	R Parietal lobe	+	-	CSE

CSE= complete surgical Excision; DA= Dural attachment, L= Left; NA=Non-available; 6MP=6 mercaptopurine, MTX= methotrexate, PSE: Partial surgical excision; PG= polyclonal gammopathy, R= Right; RIX= Rituximab; RT= radiotherapy

Out of the retrieved 42 articles on intracranial Inflammatory Myofibroblastic Tumors, 21 articles (the maximum) were published in 2001-2010, while 6 of them (the least number) were published in the decade 1980-1990. The total number of cases reported in the 42 articles was 56. Out of these 56 cases, the variables studied could not be accessed in 07 of the cases; hence data of the remaining 49 cases was recorded and analyzed.

It was observed that the cases were distributed over a wide range of ages ranging from 11 to 72 years. The average age of presentation was 29 years in the decade of 1980-1990 and 57 years in the decade of 2010-2020. Out of the 49 studied cases, 22 were males, and 27 were females (male to female ratio of 0.8:1). Headache was the most common presenting complaint seen in 18 (36%) cases, followed by seizures in 14 (28%) cases and visual disturbances in 8 (16%) cases. Other symptoms included hemiparesis, gait abnormalities, cranial nerve palsies, and hearing loss.

Concerning the lesion sites, the most common location was found to be the frontal lobe in 9 cases (18%), followed by the temporal lobe in 6 (12%) cases, the parietal lobe in 4(8%) cases, followed by 3 cases (6%) in the cavernous sinus and 2 (4%) cases in the cerebellopontine angle, and 02 (4%) cases showed multifocal intracranial involvement.

The dural attachment was noted in 22 (45%) cases and was absent in 11 (20%) cases, while it was not mentioned in the remaining. The condition was managed by complete surgical excision in 21 (43%) cases, subtotal surgical excision followed by steroid therapy in 16 (32%) cases, and radiotherapy in 11(22%) cases.

## DISCUSSION

Intracranial IMT (IIMT) is a rare clinical entity with 42 published reports since the first reported case in the year 1980. An interesting fact about IIMT is the striking resemblance with meningioma, concerning the clinical and neuroimaging profiles. This fact, along with the rarity of this entity, in the intracranial location, leads to the lack of clinical suspicion and a misdiagnosis. A study by Bradsma et al.^21^ showed how the two cases of IIMT were misdiagnosed as non-Hodgkin Lymphoma and Tuberculosis.

Although benign, the lesion maintains a progressive growth and expands in size. While most cases recover after complete surgical excision, others may require adjuvant radiotherapy. In the intracranial location, this lesion may disrupt the critical areas of the brain, causing serious complications like visual problems, nerve palsies, and hemiparesis. Hence, an early diagnosis and management are key to manage time and effectively these patients.

IIMT can present in all age groups and has been almost equally reported in males and females. Headache is the most common presenting symptom, and the frontal lobe is the most common intracranial site. Dural attachment is noted in many cases. Mostly unifocal; however, multifocal lesions are also noted. In the study by M Kilinc et al.,^3^ multiple intracranial and spinal sites were involved. Multiple intracranial sites were also found to be involved in a study by M Guduk et al.^4^

The complete surgical excision of the lesion has been found to provide good results. In a study by M Guduk et al.,^4^ IIMT involving multiple intracranial sites was managed with complete excision of all the lesions, and no recurrence was reported.

Incomplete excision of the lesion, on the other hand, was found to be associated with recurrence and may require radiotherapy. In a case study by JJ Renfrow,^36^ relapsing and recurring lesions of IIMT were encountered. This study explored the erythrocyte sedimentation rate (ESR) as a tool for monitoring the recurrence of IIMT.

Some cases also showed a locally aggressive nature. In a study by A Fukunaga,^6^ an aggressive nature of the lesion was evidenced by its spread from extra to intracranial location. Osteal erosion was reported associated with IIMT in a study by AM Buccoliero et al.,^23^ while relapsing perichondritis was found in a study by K Sato.^27^

Special histological features have been brought out in cases of IIMT in the form of necrosis,^10^ extensive ossification,^25^ and multiloculated cystic changes.^26^

Although metastasis has not been reported, cases with initial involvement in an extracranial site (lung) have been reported. In a case reported by Greiner et al.^19^ the lesion involved the lung followed by the brain involvement four years later.

Polyclonal gammopathy was noted associated with intracranial PSGs in studies by SG West^2^ and D Figarella et al.,^24^ while association with EBV was noted in the study by A Fukunaga.^6^ The association with IGG4 was found in 2 out of 4 cases by D Chen^30^ and in one case by Forcucci et al.^37^ ALK 1 was studied by D Chen^30^ in four cases and was negative.

Although surgical excision, steroid therapy, and radiotherapy have been the mainstay of treatment of IIMTS, other modalities like mercaptopurine (6MP)^40^ or Rituximab^35^ has also been tried in some cases.

## CONCLUSION

IIMT is a rare pathological diagnosis. Timely management of cases of IIMTs is crucial to reduce morbidity. To rightly diagnose IIMTs, one needs to have a high index of suspicion as these cases are likely to be misdiagnosed as meningioma on neuroimaging. Complete surgical excision should be attempted while the lesion is small to prevent a recurrence. The addition of more cases to the literature, with their associated features, will help us in further improving the current understanding of IIMTs.

## References

[1] Bahadori M, Liebow A (1973). Inflammatory Myofibroblastic Tumors of the lung. Cancer.

[2] West S, Pittman D, Coggin J (1980). Intracranial Inflammatory Myofibroblastic Tumor. Cancer.

[3] Kılınç M, Ertürk İÖ, Uysal H, Birler K, Evrenkaya T, Akkalyoncu B (2002). Multiple Inflammatory Myofibroblastic Tumor of the central nervous system: A unique case with brain and spinal cord involvement. Case report and review of literature. Spinal Cord.

[4] Güdük M, Yener U, Sav A, Pamir MN (2016). Intracranial multifocal Inflammatory Myofibroblastic Tumor: a case with multiple operations without recurrence of surgically removed lesions. Acta Neurochir (Wien).

[5] Figarella-Branger D, Gambarelli D, Perez-Castillo M, Garbe L, Grisoli F (1990). Primary Intracerebral Inflammatory Myofibroblastic Tumor: A Light, Immunocytochemical, and ultrastructural study of one case. Neurosurgery.

[6] Fukunaga A, Yoshida K, Otani M (1998). Inflammatory Myofibroblastic Tumor extending from the extracranial to the intracranial space associated with Epstein-Barr virus infection — Case report. Neurol Med Chir (Tokyo).

[7] Cannella D, Prezyna A, Kapp J (1988). Primary intracranial plasma-cell granuloma. J Neurosurg.

[8] Gangemi M, Maiuri F, Giamundo A, Donati P, De Chiara A. (1989). Intracranial Inflammatory Myofibroblastic Tumor. Neurosurgery.

[9] Maeda Y, Tani E, Nakano M, Matsumoto T (1984). Plasma-cell granuloma of the fourth ventricle. J Neurosurg.

[10] Gochman GA, Duffy K, Crandall PH, Vinters HV (1990). Inflammatory Myofibroblastic Tumor of the brain. Surg Neurol.

[11] Makino K, Murakami M, Kitano I, Ushio Y (1995). Primary intracranial plasma-cell granuloma: A case report and review of the literature. Surg Neurol.

[12] Le Marc’hadour F, Fransen P, Labat-Moleur F, Passagia JG, Pasquier B (1994). Intracranial Inflammatory Myofibroblastic Tumor: A report of four cases. Surg Neurol.

[13] Breidahl W, Robbins P, Ives F, Wong G (1996). Intracranial Inflammatory Myofibroblastic Tumor. Neuroradiology.

[14] Dettmeyer R, Varchmin-Schultheiß K, Madea B (1998). Intracranial Inflammatory Myofibroblastic Tumor and Homocystinuria. Pathol Res Pract.

[15] Saxena A, Sinha S, Tatke M (2000). Intracranial Inflammatory Myofibroblastic Tumor ‘; a case report and review of the literature. Br J Neurosurg.

[16] Tekkök I, Ventureyra E, Jimenez C (2000). Intracranial Inflammatory Myofibroblastic Tumor. Brain Tumor Pathol.

[17] Murakami K, Muraishi K, Ikeda H, Yoshimoto T (2001). Inflammatory Myofibroblastic Tumor of the pituitary gland. Surg Neurol.

[18] Lyo I, Suh J, Kwon Y (2001). Intracranial Inflammatory Myofibroblastic Tumor: A Case Report. J Korean Neurosurg Soc.

[19] Greiner C, Rickert CH, Möllmann FT (2003). Inflammatory Myofibroblastic Tumor involving the brain and the lung. Acta Neurochir (Wien).

[20] Choi CY, Whang CJ, Park SH (2003). Intracranial Inflammatory Myofibroblastic Tumor. J Korean Neurosurg Soc.

[21] Brandsma D, Jansen G, Spliet W, van Nielen K, Taphoorn M (2003). The diagnostic difficulties of meningeal and intracerebral Inflammatory Myofibroblastic Tumors - Presentation of three cases. J Neurol.

[22] Murakami M, Hashimoto N, Kimura S, Hosokawa Y, Kakita K (2003). Intracranial Inflammatory Myofibroblastic Tumor with genetic analysis. Acta Neurochir (Wien).

[23] Buccoliero A, Caldarella A, Santucci M, Ammannati F, Mennonna P, Taddei A (2003). Inflammatory Myofibroblastic Tumor—An enigmatic lesion: Description of an extensive intracranial case and review of the literature. Arch Pathol Lab Med.

[24] Roche P-H, Figarella-Branger D, Pellet W (2004). Mixed meningeal and brain plasma-cell granuloma: an example of an unusual evolution. Acta Neurochir (Wien).

[25] Özüm Ü, Özer H, Karadağ Ö, Polat N (2006). Intracranial plasma cell-granuloma with extensive ossification. Br J Neurosurg.

[26] Nawashiro H, Omura T, Kobayashi H (2006). Cystic Intracranial Inflammatory Myofibroblastic Tumor. J Neurosurg.

[27] Sato K, Kubota T, Kitai R, Miyamori I (2006). Meningeal Inflammatory Myofibroblastic Tumor with relapsing polychondritis. J Neurosurg.

[28] Flannery T, Al-Sabah F, Bhangu J, Alderazi Y, Brett F, Pidgeon C (2007). Treatment of subtotally resected intracranial Inflammatory Myofibroblastic Tumor with steroids: a case report. Br J Neurosurg.

[29] Kim D-J, Choi Y-S, Song Y-J, Kim K-U (2009). Intracranial Inflammatory Myofibroblastic Tumor. J Korean Neurosurg Soc.

[30] Chen D, Liu L, Qiu L (2009). The MRI Misdiagnosis Analysis of Intracranial Inflammatory Myofibroblastic Tumor (4 Cases Report and Literature Review). Journal of Clinical Radiology.

[31] Agarwal A, Aronov R, Agarwal K, Lee HK, Zak I, Kish K (2010). Inflammatory Myofibroblastic Tumor of the brain: an unusual case. Eur J Radiol Extra.

[32] Cao X, Luan S, Sun L, Yang B, Shen C, Bao W (2010). Impaired vision associated with a solitary intracranial plasmacytoma. J Clin Neurosci.

[33] Brito C, Lopes F, Chimelli L, Gasparetto E (2010). Intracranial cell plasma granuloma. Arq Neuropsiquiatr.

[34] Puntambekar P, Santhakumar S, Kupsky WJ, Tselis A, Mittal S (2011). Primary intracranial Inflammatory Myofibroblastic Tumors presenting as malignant neoplasms. J Neurooncol.

[35] Schneider C, Henning T, Fink G, Schroeter M, Lehmann H (2014). Primary intracranial Inflammatory Myofibroblastic Tumor responsive to rituximab. Neurology.

[36] Renfrow JJ, Mitchell JW, Goodman M (2013). Relapsing intracranial Inflammatory Myofibroblastic Tumor: A case report. Oncol Lett.

[37] Forcucci J, Butler-Williams S, Miller N, Lazarchick J (2015). Inflammatory Myofibroblastic Tumor: An n9entity within the spectrum of IgG4-related disease. Ann Clin Lab Sci.

[38] Bibars W, Singh H, Zbytek B. (2016). Inflammatory Myofibroblastic Tumor in Cerebellum: A case report. Am. J. Clin. Pathol.

[39] Gautam S, Ramesh V, Karthikeyan K, Krishnakumar M (2017). Intracranial inflammatory pseudotumor presenting as an en plaque mass. Neurol India.

[40] Shah M, McClain K (2005). Intracranial Inflammatory Myofibroblastic Tumor. J Pediatr Hematol Oncol.

